# Dew Point Calibration System Using a Quartz Crystal Sensor with a Differential Frequency Method

**DOI:** 10.3390/s16111944

**Published:** 2016-11-18

**Authors:** Ningning Lin, Xiaofeng Meng, Jing Nie

**Affiliations:** Science and Technology on Inertial Laboratory, Beihang University, Beijing 100191, China; linningning@buaa.edu.cn (N.L.); mengxf@buaa.edu.cn (X.M.)

**Keywords:** differential frequency, dew point, quartz crystal sensor, spectrum analysis

## Abstract

In this paper, the influence of temperature on quartz crystal microbalance (QCM) sensor response during dew point calibration is investigated. The aim is to present a compensation method to eliminate temperature impact on frequency acquisition. A new sensitive structure is proposed with double QCMs. One is kept in contact with the environment, whereas the other is not exposed to the atmosphere. There is a thermal conductivity silicone pad between each crystal and a refrigeration device to keep a uniform temperature condition. A differential frequency method is described in detail and is applied to calibrate the frequency characteristics of QCM at the dew point of −3.75 °C. It is worth noting that frequency changes of two QCMs were approximately opposite when temperature conditions were changed simultaneously. The results from continuous experiments show that the frequencies of two QCMs as the dew point moment was reached have strong consistency and high repeatability, leading to the conclusion that the sensitive structure can calibrate dew points with high reliability.

## 1. Introduction

Precision monitoring on ambient humidity plays a crucial role in myriad automatic production such as in electronics, machinery, agriculture, pharmacy, and storage [1]. The key task is to develop humidity sensors possessing high sensitivity and rapid response. In recent years, many efforts have focused on advanced-humidity sensor-related research [2,3,4,5]. Dew point hygrometers have been used as a standard for measuring the humidity of gases and are used in many laboratories as the reference calibration standard. Many different dew point hygrometers based on chilled mirrors [6], surface acoustic waves [7], optical fibers [8], microwave resonators [9], CMOS technology with capacitive detection of condensed water, [10] etc., have been reported.

The most popular mass sensor, known as a quartz crystal microbalance (QCM), is a very stable and sensitive device, and its applications have extended to biochemistry, environmental monitoring, explosives detection, and intrusion detection [11,12,13]. Recently, the QCM has been used as a humidity sensor [14,15,16,17]. One of the potential problems about QCM-based sensors is their cross sensitivity to thermal fluctuations, which can seriously compromise sensor accuracy [18,19,20,21]. It is a common issue in applications where the QCM is used for in situ sensor measurements in dynamically variable environments [21,22], where the sensor often operates over a wide temperature range. In such situations, the sensor readings will be superimposed with the QCM’s temperature-induced frequency shifts, and this will cause measurement errors [21]. Therefore, temperature has become an important error source in the process of dew point calibration. 

Our team has focused on the QCM dew-point sensor. In our previous work, the sensor is used to detect the dew point at the range of 60%–95% RH [23]. Unfortunately, the dew errors caused by temperature have not been completely resolved. To solve the problem that resonance frequency of the quartz is very sensitive to thermal changes, plenty of studies have been done. The main solutions include selecting an appropriate angle of crystal cut for every desired temperature range, to compensate for drift using analog or digital voltage compensation, to use an additional reference crystal, and to use baseline subtraction [18]. In one study [24] a frequency–temperature characteristic curve in dry air (3%) and then a frequency characteristic curve counteracting the effect of temperature were obtained. However, the temperature of the crystal sensor must be monitored accurately without significant noise or spatial and temporal hysteresis. Making the same measurement on an array of sensors that have been treated to yield diverse functional behaviors is a quite suitable option for continuous and online monitoring [25,26], and a better consideration of structure is needed. In another study [27] a dual quartz crystal microbalance (DQCM) using the photolithographic method was developed. Other research [28] has involved the construction of a new structure whereby two crystals with slightly different frequencies were laminated together to form an air-gap sandwich, and it was found that “cross-talk” between the two crystals did not occur, which provides some edification with respect to the design of a DQCM.

In this paper, a method is proposed to remove the temperature impact on dew point calibration by proposing a new, sensitive structure based on DQCM. The sensor combines configurational advantages with a rapid response time for low humidity. Two crystals are stimulated in turns by a relay, and their frequencies are acquired through real-time monitoring based on the spectrum analysis method. Additionally, two crystals are cooled by a refrigeration device simultaneously, so that the frequency change induced by the temperature can be easily eliminated. Experiments were also conducted at this dew point of −3.75 °C. The frequency results have strong consistency and high repeatability, proving that this sensitive structure can calibrate dew point with high reliability.

## 2. Materials and Methods

### 2.1. Sensitive Structure

**Figure 1:**
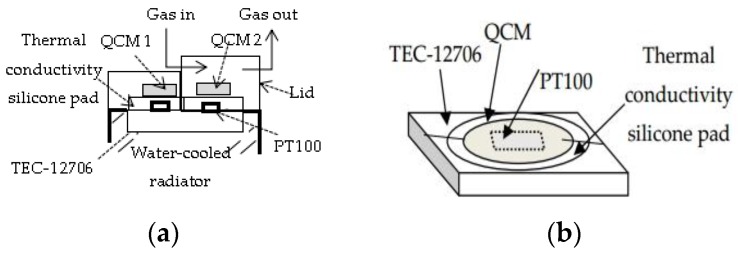
The sensitive structure: (**a**) top view and front view; (**b**) sensor diagram.

As shown in Figure 1a,b, the core of the assembled sensitive structure consists of two QCM wafers. They are 13.98 mm in diameter with 13.98-mm-diameter outer and 6.0-mm-diameter inner electrodes. Slightly different frequencies were chosen for the two crystals in each probe to minimize internal interference. Two PT100 temperature sensors were glued onto the cold surface of a refrigeration device. Unlike mounting a copper metallic O-ring [15] or spring [29], two circles of thermal conductive silicone pads are stuck along the outer edge of the PT100 sensors, and the outer edge of the DQCM is bonded to the thermal conductive silicone pads to seal the lower surface, which benefits the uniformity of the heat transfer and shortens the response time of the dew point. Moreover, the hot surface of the semiconductor refrigerator piece is pasted to a water-cooled radiator to ensure more efficient refrigeration. Two electrode leads of each QCM are fixed in the outside and connected to a frequency sweep generator through a relay. After wiring and testing, QCM2 and QCM1 are covered by lids with and without breather pipes, respectively. The lid made from Teflon is fastened with screws externally. A gas channel passes through the upper surface of QCM2, yet there is no gas exchange with the external environment for QCM1. Considering that little remaining gas still exists on the upper surface of QCM1, extremely dry gas with a humidity of 3% is pumped into a hole that was drilled through the lid at the start of the measurement so that water condensation will not appear on the upper surface of QCM1 in a large temperature range.

The sensitive structure possesses configurational advantages. One significant configurational feature is the consistency of both crystals. Two crystals are cooled by a refrigeration device simultaneously in order to expose them to the same temperature environment. As a result, the frequency difference of the DQCM calculated by the adjacent time can fully get rid of the frequency changes induced by temperature. Another feature is that the isolation of both excited electrodes on each crystal by bonding the periphery of the lower surface to a thermal conductive silicone pad. This prevents water condensation on both surfaces of the crystal and thus secures an improved resonance. 

QCM1 is out of contact with the measured gas environment as a reference crystal, and its frequency is denoted by *f_s_*, while QCM2 is in contact with the measured gas environment as a work crystal, and its frequency is written as *f_d_*. The refrigeration device controls the temperature on the lower surface of the DQCM driven by an external current and leads to the condensation of water molecules. The mass change on the upper surface of QCM2 thus leads to the variation in resonance frequency. However, the frequency offset is partially caused by temperature variation. On account of the fact that no mass deposition occurs on QCM1, the frequency difference of DQCM can adequately eliminate the temperature effect. The measured temperature when water molecules congeal on the surface of QCM2 corresponds to the dew point. Therefore, it is reasonable to achieve recognition of the dew point by measuring the frequency characteristic of the DQCM. Moreover, because the switch time of the relay is only 3 ms, and the measuring time of each frequency is within 1 s, the time difference between *f_s_* and *f_d_* can be ignored. Meanwhile, when the temperature changes slowly enough, the frequencies of the two crystals in turn can be regarded as being measured in the same temperature condition. As a result, the differential (heterodyned) frequency measurement substantially eliminates the deleterious effect of the temperature, and the accuracy of the dew point calibration can be improved accordingly. In addition, fast frequency measurement within 1 s enhances the real-time performance of dew point calibration and reflects the phase change process well. A continuous monitoring in a closed-loop system for a constant dew point helps to reduce random error so that the dew point is captured more accurately.

### 2.2. Experimental Setup

**Figure 2:**
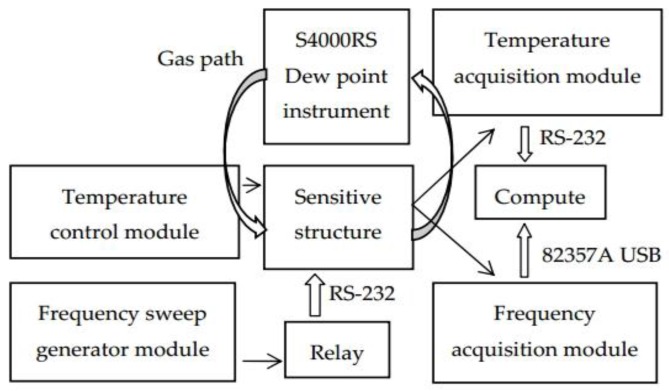
Experimental set-up.

The experimental setup is depicted in Figure 2.

The DQCM in the sensitive structure is made up of two AT-cut quartz crystals provided by TAITIEN, USA (S-DBAAB-6MK01), with the fundamental resonance frequencies of 5.989 and 5.987 MHz, respectively. 

An Agilent 4294A Precision Impedance Analyzer was chosen as the frequency sweep generator module as well as the frequency acquisition module. A relay is linked to the impedance analyzer for alternate scanning frequencies of the DQCM. A personal computer is connected to the frequency acquisition module via an Agilent 82357A USB interface.

A temperature control module exports analog voltage with a data acquisition card (PCI1716) at first, and the analog voltage then turns into an analog current via a power amplification circuit. Thus, we control the cooling power of the refrigeration device with an adjustable input current. The temperature acquisition module uses two temperature transducers—PT100 with a three-wire system. The analog signal is transferred to a digital signal via ADAM-4015 and ADAM-4520, and the signal is displayed on the computer. 

Furthermore, the S4000RS dew point instrument is in the same humidity environment as QCM2 by air tubes in parallel, and the output of the S4000RS dew point instrument with a measurement error of ± 0.01°C is regarded as the reference dew point, which is denoted by *T*_0_. 

### 2.3. Spectrum Analysis Method

The sensitive structure requires a rapid response while the resonance frequency change in the temperature control period is tracked. Spectrum analysis is one of the methods by which the frequency of the QCM is detected. A QCM is stimulated for the oscillation with a sweep signal, so the resonance frequency of the QCM can be detected via scanning. We continuously measure the resonance frequencies of the DQCM with an Agilent 4294A Precision Impedance Analyzer, which has the function of a broadband sweeping with a frequency resolution of 10^−6^ in the frequency band. The resonance frequencies are determined from the maximum of the conductance peaks, which correspond to the electrical series resonance frequencies (MSRFs) of the equivalent Butterworth–van Dyke (BVD) circuit [30].

There are two means of calculation frequency. The electrical parameters are measured using an equivalent BVD circuit model of quartz crystal by Agilent4294A Precision Impedance Analyzer and are placed into Equation (1), and the resonance frequency of QCM can be thus be calculated in about 2–3 s [31].

(1) $$f_{0}=\frac{1}{2\pi \sqrt{L_{q}C_{q}}}$$

where $f_{0}$ represents series resonance frequency, and the inductance and capacitance in BVD equivalent circuit model of quartz crystal are denoted by $L_{q}$ and $C_{q}$ respectively.

Secondly, the resonance frequency of QCM can also be calculated through the admittance characteristics of the quartz crystal. The admittance Y(jw) is expressed as follows [31]:

(2) $$Y(jw)=G+jB=\frac{1}{R_{q}+jwL_{q}+\frac{1}{jwC_{q}}}+jwC_{0}.$$

The real and imaginary part of admittance is written as *G* and *B*, respectively, to obtain Equation (3).

(3) $${\left(G-\frac{1}{2R_{q}}\right)}^{2}+{\left(B-wC_{q}\right)}^{2}={\left(\frac{1}{2R_{q}}\right)}^{2}$$

where $R_{q}$ represents the MSRF, and *f * is represents the measured frequency points in the given frequency band in w=2πf. *G* obtains a peak when scanning frequency equals $f_{0}$ and $G_{\max}=\frac{1}{R_{q}}$.

### 2.4. Function Realization of Software

A real-time monitor computer program is executed with LabVIEW, which is based on the second method mentioned in Section 2.3. It achieved a once frequency measuring time within 1 s with an impedance analyzer, as well as frequency resolution of 1 Hz. Therefore, the second method applied to our computer program, compared with the first method, has a faster speed of frequency measurement, which benefits the real-time tracking of the dew point. Within each frequency measuring time, multi-point impedance amplitudes and frequencies near the resonance frequency $f_{0}$ of the crystal were measured, and $f_{0}$ was obtained at the minimum impedance amplitude.

An impedance instrument is used for principle analysis in most cases, but here it is used for the real-time detection of resonance frequency. Frequency resolution *γ* is calculated via Equation (4):

*γ* = *δ*/*n* (4)

where *δ* represents the range of sweeping frequency, and *n* stands for the scanning points. The key factor of sweep time for $f_{0}$ is the scanning points, and the greater *n* is, the more sweep time for $f_{0}$ it spends. If we reduce *n*, the frequency resolution will increase accordingly in the constant frequency sweep interval, which results in poor frequency accuracy. Hence, although it is better to measure $f_{0}$ quickly, we have to comprehensively weigh the sweep time for $f_{0}$ and the frequency measuring accuracy.

**Figure 3:**
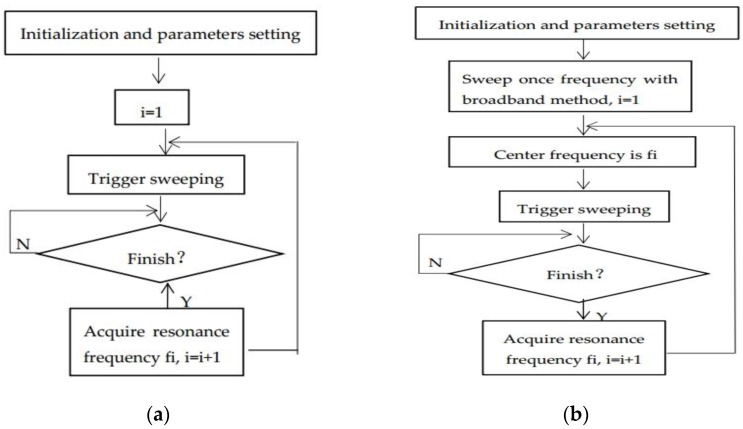
Two methods of measuring $f_{0}$: (**a**) the broadband method and (**b**) the narrow band method.

An impedance instrument has the function of a broadband frequency sweep, so the direct method of frequency measuring is a broadband method. A basic flow chart is shown in Figure 3a. In order to secure the largest range of sweeping frequency, *δ* must be properly set. However, over the long period of continuous measurement, it is difficult to master an accurate range, which leads to an inappropriate setting of the frequency’s bounds. There are two consequences to this: One is that, if *δ* is too narrow to cover the resonance frequency, the output frequency will be the upper or lower bound, and an error will occur. The other is that *δ* is set large enough to cover the resonance frequency, and *γ* may increase at the expense of extending the measuring time. In addition, a new frequency measuring method is put forward, which is called the narrow band method. The basic flow chart is as shown in Figure 3b. The approach of seeking the resonance frequency is that, firstly, with the broadband method, we sweep $f_{0}$ once and set it as the initial center frequency of sweeping. Then, in the loop measurement, center frequency will be replaced by each new measured $f_{0}$.

**Figure 4:**
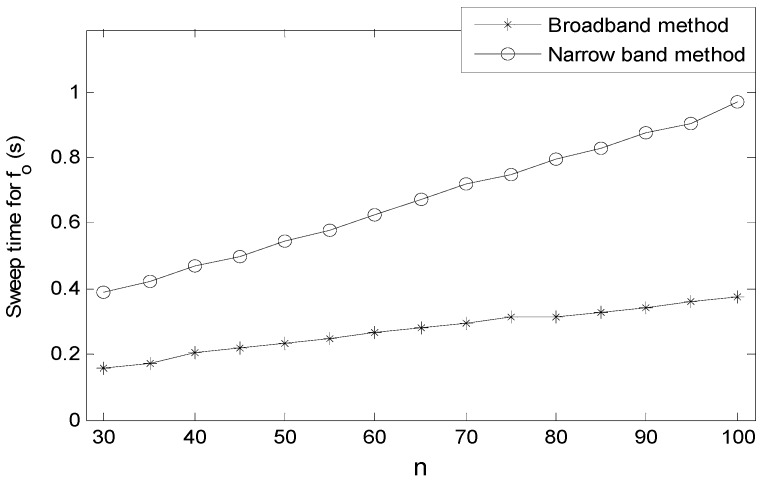
The relationship between *n *and sweep time for $f_{0}$.

As for Agilent 4294A, when $f_{0}$ > 500 kHz and measurement bandwidth is set to 1 dB, the impedance of each frequency point is measured in 3 ms. Sweep time for $f_{0}$ must be determined via experiment because there is a communication delay between computer and impedance instrument. The relationship between *n* and sweep time for $f_{0}$ is described in Figure 4. It is obvious that sweep time for $f_{0}$ increases linearly as the augment of *n* in two ways. *n* limited in 100 meets the requirement of dew point calibration; therefore, sweep time for $f_{0}$ is within 1 s.

The narrow band method has an apparent advantage compared with the broadband method. Once measuring is finished, and the center frequency will update. Therefore, the range of sweeping frequency virtually increases over the entire measurement process, and we avoid the problem of difficulty in the setting range of sweeping frequency. Because the frequency variation of the QCM is a graded parameter during the condensation, we can achieve frequency tracking effectively with this method.

## 3. Experiments and Results

**Figure 5:**
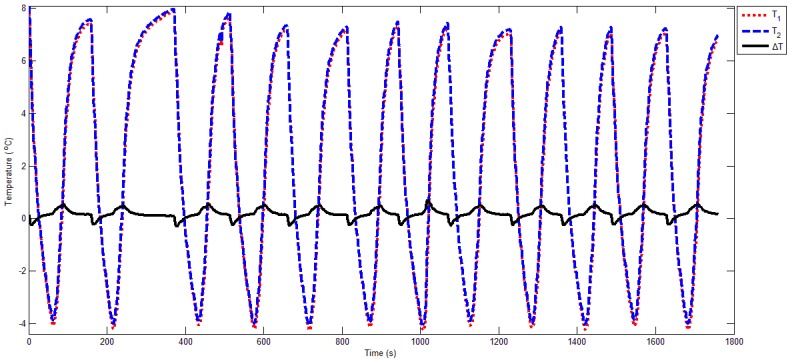
The measured temperature of the dual quartz crystal microbalance (DQCM).

QCM1 is the reference crystal, QCM2 is the work crystal, and the measured temperatures via the two PT100 sensors are written as *T*_1_ and *T*_2_, respectively. Though the DQCM are cooled by a refrigeration device (TEC1-12706), the temperature gradient causes a temperature difference, denoted by Δ*T*. Figure 5 indicates that Δ*T* is within 0.5 °C; thus, the controlling temperature of the DQCM can be considered as constant. We took the average of *T*_1_ and *T*_2_ and named it *T*, representing the measured temperature. During each cooling period, we aimed to recognize the moment at which *T* and *T*_0_ were equal; at this point, Δ*f1 = f_s_ − f_d_* reflected the frequency characteristic.

**Figure 6:**
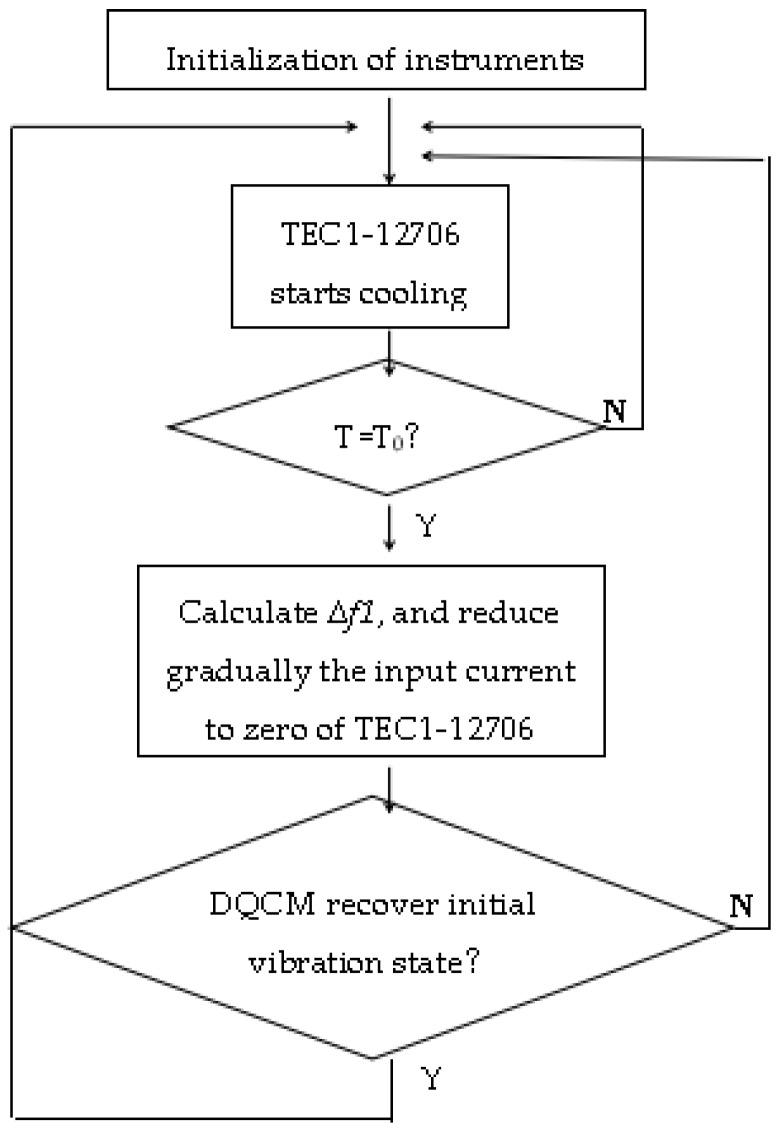
Work flow chart.

The purpose of continuous dew point calibration is to observe the relationship between the frequency difference of the DQCM and the dew point in many temperature circles. When *T = T*_0_, TEC1-12706 stops cooling, and it does not start cooling until the DQCM recovers the initial vibration state. The narrow band method mentioned above is adopted, and the frequencies of the DQCM are monitored in real time. The frequency difference of QCM1 and QCM2 is written as Δ*f*, which is calculated by Δ*f = f_s_* *− f_d_*, where Δ*f1* represents the frequency difference at the dew point moment and Δ*f2* stands for the frequency difference when the DQCM recovers the initial vibration state. A work flow chart is shown in Figure 6.

**Figure 7:**
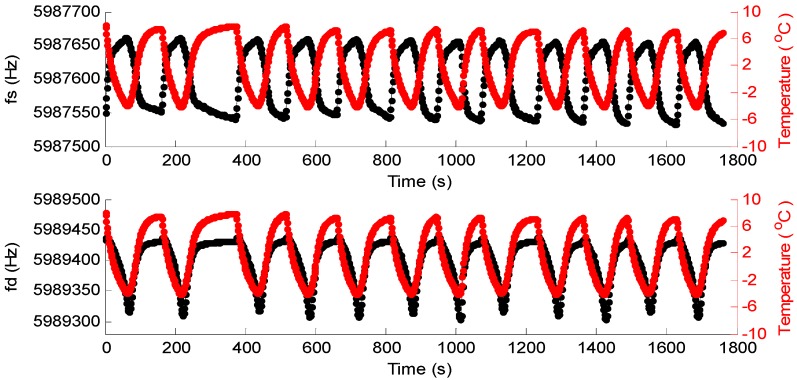
The frequencies of the DQCM with the change in temperature.

**Figure 8:**
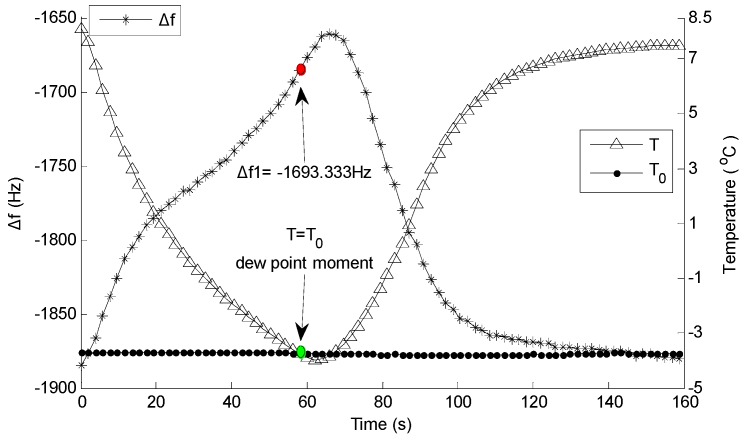
Variation regularity of Δ*f* in a temperature-control circle.

The dew point of the tested gas was set at −3.75 °C, and pumped the tested gas for 10 min into the system to make sure that QCM2 was in an environment of uniform humidity. The water temperature in the water-cooled radiator was set at 5 °C in order to shorten the cooling time. Figure 7 shows the frequencies of the DQCM with the change in temperature; meanwhile, Δ*f* in a temperature-control circle was calculated and recorded (Figure 8).

Figure 7 shows that the frequencies of the DQCM have a certain regularity with the change in temperature. On the one hand, the frequency of QCM1 increased gradually as temperature dropped, and decreased as temperature rose, but the overall range was within 120 Hz. On the other hand, the changing regularity of QCM2’s frequency was approximately the opposite. The frequency of QCM2 slightly increased and then decreased as temperature dropped, increased gradually as temperature rose, and then returned to the initial frequency. Figure 8 indicates that, Δ*f* increased gradually as temperature dropped, and decreased as temperature rose. The point highlighted in green is the moment of *T = T*_0_, namely, the dew point moment, and the red dot represents Δ*f* at that time, which is noted by Δ*f1*. The calculated Δ*f* in 11 groups of temperature cycles are listed in Table 1; the variation in the DQCM’s frequency difference as the dew point moment is reached, which is denoted by *df*, was calculated by *df = |*Δ*f1 − *Δ*f2|.*

Since the frequency difference affected by the interference of temperature is canceled, *df* is merely attributable to water condensation on the upper surface of the QCM. It can be inferred from Table 1 that *df* is virtually a fixed value, and its value is *df* = 191.416 ± 6.566 Hz.

It can be concluded that *df* has excellent reproducibility in the 11 groups, which proves that the system has good repeatability for dew point calibration.

As a result, if only one QCM is used to calibrate the dew point, the frequency characteristic will contain the part impacted by temperature, which results in an error. It further reflects the necessity of temperature compensation during the process of dew point calibration.

## 4. Conclusions

It is obvious that temperature is a factor that affects the precision of frequency characteristics. In this paper, we use a differential frequency method with a DQCM for temperature compensation, and effectively offset this effect. A host computer program is executed that achieved a once frequency measuring time within 1 s with an impedance analyzer, as well as a frequency resolution of 1 Hz. Therefore, the real-time performance of frequency measurement was achieved, and the measurement reflects the phase change process well. It is worth mentioning that, as DQCMs are cooled by TEC1-12706 with a similar temperature, this sensitive structure can overcome limitations that need to strictly maintain a consistent changing process of temperature. Under a fixed humidity environment, the variation in the DQCM’s frequency difference as the dew point moment is reached is nearly constant and demonstrates the amount of electrode surface condensation, clearly demonstrating that this test system has good repeatability. Thus, the differential frequency method with a DQCM has is significant in the development of QCM sensors in the liquid phase, providing foundations for the design of a new on-line QCM sensor with high accuracy.

## Figures and Tables

**Table 1:** Δ*f* in 11 groups of temperature cycles.

Δ*f1* (Hz)	Δ*f2* (Hz)	*df* (Hz)
−1693.333	−1880.200	186.867
−1695.349	−1880.200	184.851
−1694.338	−1891.306	196.968
−1696.354	−1885.244	188.890
−1697.363	−1890.293	192.930
−1701.399	−1892.310	190.911
−1695.337	−1892.310	196.973
−1695.333	−1893.311	197.978
−1698.361	−1893.311	194.950
−1698.358	−1895.327	196.969
−1700.373	−1898.355	197.982
